# Progranulin in Musculoskeletal Inflammatory and Degenerative Disorders, Focus on Rheumatoid Arthritis, Lupus and Intervertebral Disc Disease: A Systematic Review

**DOI:** 10.3390/ph15121544

**Published:** 2022-12-12

**Authors:** María González-Rodríguez, Djedjiga Ait Edjoudi, Alfonso Cordero Barreal, Clara Ruiz-Fernández, Mariam Farrag, Beatriz González-Rodríguez, Francisca Lago, Maurizio Capuozzo, Miguel Angel Gonzalez-Gay, Antonio Mera Varela, Jesús Pino, Yousof Farrag, Oreste Gualillo

**Affiliations:** 1 SERGAS (Servizo Galego de Saude), and IDIS (Instituto de Investigación Sanitaria de Santiago), NEIRID Lab (Neuroendocrine Interactions in Rheumatology and Inflammatory Diseases), Research Laboratory 9, Santiago University Clinical Hospital, 15706 Santiago de Compostela, Spain; 2 International PhD School, University of Santiago de Compostela (EDIUS), 15706 Santiago de Compostela, Spain; 3 SESCAM (Servicio de Salud de Castilla La Mancha), Ophthalmology Department, University Hospital of Toledo, 45007 Toledo, Spain; 4 Molecular and Cellular Cardiology Group, SERGAS (Servizo Galego de Saude), and IDIS (Instituto de Investigación Sanitaria de Santiago), Research Laboratory 7, Santiago University Clinical Hospital, 15706 Santiago de Compostela, Spain; 5 National Health Service, Local Health Authority ASL 3 Napoli Sud, Department of Pharmacy, 80056 Naples, Italy; 6 Hospital Universitario Marqués de Valdecilla, Epidemiology, Genetics and Atherosclerosis Research Group on Systemic Inflammatory Diseases, IDIVAL, University of Cantabria, Avenida de Valdecilla s/n, 39008 Santander, Spain; 7 SERGAS, Servizo Galego de Saude, Santiago University Clinical Hospital, Division of Rheumatology, 15706 Santiago de Compostela, Spain

**Keywords:** progranulin, inflammatory diseases, rheumatoid arthritis, lupus, intervertebral disc disease

## Abstract

Progranulin (PGRN) is a glycoprotein formed by 593 amino acids encoded by the GRN gene. It has an important role in immunity and inflammatory response, as well as in tissue recovery. Its role in musculoskeletal inflammatory diseases such as rheumatoid arthritis (RA), systemic lupus erythematosus (SLE) and intervertebral disc degeneration disease (IVDD), is, nowadays, an important target to investigate. The objective of this review is to systematically sum up all the recent findings concerning PGRN as a target in the development and resolution of the inflammatory diseases. PubMed was examined with the terms combinations (Progranulin) AND (Lupus Erythematosus, Systemic), (Progranulin) AND (Arthritis, Rheumatoid), and (Progranulin) AND (Intervertebral Disc Degeneration). PubMed was examined with the terms combinations (Atsttrin) AND (Lupus Erythematosus, Systemic), (Atsttrin) AND (Arthritis, Rheumatoid), and (Atsttrin) AND (Intervertebral Disc Degeneration). Moreover, research through Web of Science was performed searching the same items. The inclusion criteria were: studies whose main topic were progranulin, or atsttrin, with emphasis on the three selected diseases. On the other hand, the exclusion criteria were studies that only focused on diseases not related to RA, lupus or IVDD, in addition to the previous published literature reviews. Since few results were obtained, we did not filter by year. The records assessed for eligibility were 23, including all the studies with the information in state of art of progranulin and its capability to be a potential target or treatment for each one of the selected diseases. As these results are descriptive and not clinical trials, we did not perform risk of bias methods. Within these results, many studies have shown an anti-inflammatory activity of PGRN in RA. PGRN levels in serum and synovial fluids in RA patients were reported higher than controls. On the other hand, serum levels were directly correlated with SLE disease activity index, suggesting an important role of PGRN as a player in the progression of inflammatory diseases and a therapeutical approach for the recovery. This review has some limitations due to the small number of studies in this regard; therefore, we highlight the importance and the necessity of further investigation. No external funding was implicated in this systematical review.

## 1. Introduction

Progranulin (PGRN) is an auto endocrine growth factor formed of 593 amino acids. This adipokine was extracted for the first time as a growth factor in conditioned tissue culture media [1]. PGRN is also recognized as granulin epithelin precursor (GEP), PC-cell-derived growth factor (PCDGF), proepithelin and acrogranin. It can be detected in a wide range of cells, such as: epithelial cells [2], neuronal cells [3], chondrocytes [4], adipocytes [5], macrophages, endothelial cells and skeletal muscle cells [6], which makes it a molecule that can be involved in various pathologies.

**Figure 1:**
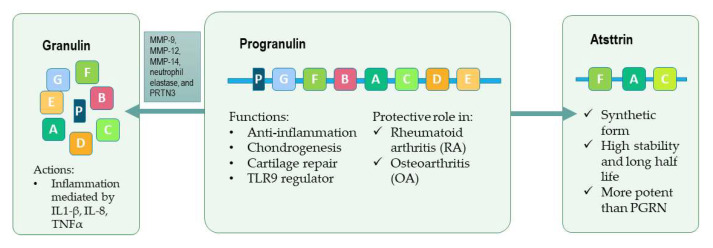
Progranulin (PGRN) structure and its derivative products. PGRN is formed by 7.5 domains of a 12-cysteine motif. Atsttrin is the synthetic form derived from FAC domain of PGRN. Elastase and other enzymes promote PGRN cleavage producing GRN, that promotes inflammation.

**Figure 2:**
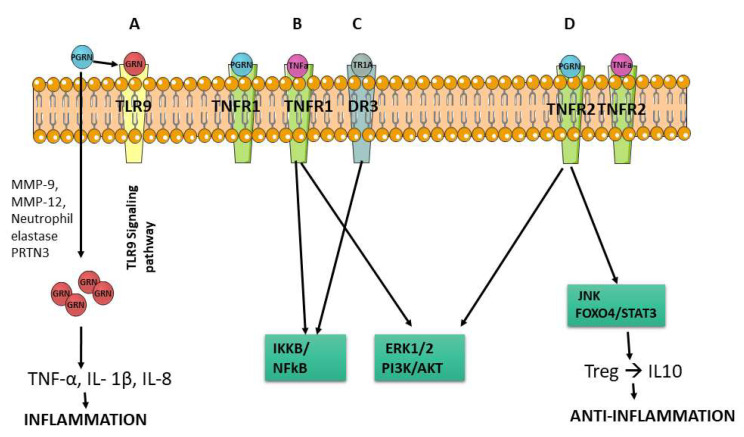
Schematic representation for the principal PGRN and granulin mechanisms. (**A**) TLR9 pathway activated by granulin, the degradation product of PRGN, releasing TNF-α, IL-1β and IL-8 causing inflammation. (**B**) The inflammatory pathways NF-κB, ERK1/2 and PI3K/AKT are activated through TNF-α binding to TNFR1. (**C**) PGRN binds to DR3, which has the highest homology to TNFR, interfering with the attachment of DR3 and TL1A. (**D**) PGRN induces the synthesis and secretion of IL-10, from Treg through JNK pathway, depending on TNFR2.

PGRN is formed of seven and a half domains of a 12-cysteine motif (CX5–6CX5CCX8CCX6CCXDX2HCCPX4CX5–6C, X = any amino acid) in the order P-G-F-B-A-C-D-E [7] (Figure 1). This structure is susceptible to proteolytic cleavage by elastase and other enzymes releasing individual granulin (GRN) units (Figure 1). Although the full length PGRN is reported to have anti-inflammatory action, the cleaved granulin units are believed to be proinflammatory and can counteract the anti-inflammatory activity of PGRN [8,9]. These GRN peptides are able to increase the expression of interleukin 1 beta (IL- 1β), IL-8, and tumor necrosis factor alpha (TNF-α) [10], and also to bind to toll-like receptor 9 (TLR9) activating its pathway (Figure 2A) [11].

During the inflammation process, neutrophils and macrophages secrete matrix metalloproteinases (MMP-9, MMP-12, MMP-14) [12,13], neutrophil elastase and proteinase 3 (PRTN3) [14]. In this context, PGRN can be degraded to 6-kDa granulin peptides that principally have an inflammatory role [15]. Apolipoprotein A1, which binds the full-length PGRN to prevent protease hydrolysis, and the secretory leukocyte protease inhibitor (SLPI), on the other hand, are necessary for the protection of the anti-inflammatory characteristics of PGRN [15,16,17].

The anti-inflammatory effect of PGRN has drawn more attention than the proinflammatory effect of its cleavage products. In order to mediate the TNF-α/TNFR signaling pathway, PGRN binds to the cysteine-rich domain 2 (CRD2) and CRD3 of the extracellular regions of TNFR1 and TNFR2 [18]. PGRN and TNF-α have the same binding affinity to TNFR1, but PGRN has an approximately 600 times higher affinity to TNFR2. To compete with the activation of the NF-κB inflammatory pathway via TNF-α, PGRN binds to TNFR1 and activates the extracellular regulated protein kinase 1 and 2 (ERK1/2) and phosphatidylinositol 3 kinases/protein kinase B (PI3K/AKT) pathways (Figure 2B) [18,19,20]. In addition, PGRN can bind to death receptor 3 (DR3), which has the highest homology to TNFR, to prevent the attaching of DR3 and TNF-like ligand 1A (TL1A), performing an additional anti-inflammatory effect (Figure 2C) [21,22].

PGRN also exerts other anti-inflammatory mechanism, leading to the synthesis and secretion of IL-10, from regulatory T lymphocytes (Treg) [23] through c-Jun N-terminal kinase pathway (JNK), depending on TNFR2 (Figure 2D). In diseases such as inflammatory arthritis, PGRN-induced IL-10 production in Treg cells depends on the forkhead box protein O4/signal transducer and activator of transcription 3 (Foxo4/STAT3) pathway [24,25].

PGRN, through TNFR1, specifically blocks the expression and the release of CXCL9 and CXCL10 triggered by TNF-α [26]. It is interesting to note that PGRN can also reduce the production of CXCL9 and CXCL10 via IFN-γ, which may be another possible mechanism for the regulation of the inflammatory process [27].

Given its pleiotropic functions and implication in inflammatory process and immune mechanisms, this adipokine could be a target for potential therapeutic approach for the treatment of arthritic and inflammatory diseases.

## 2. Results

In PubMed, from the terms (Progranulin) AND (Lupus Erythematosus, Systemic), we found 9 results from 2012 to 2022; from (Progranulin) AND (Arthritis, Rheumatoid), we found 26 results from 2009 to 2022; and from (Progranulin) AND (Intervertebral Disc Degeneration), we found 4 results from 2015 to 2022. The terms combinations (Atsttrin) AND (Lupus Erythematosus, Systemic) showed 0 results, (Atsttrin) AND (Arthritis, Rheumatoid) showed 4 results, and (Atsttrin) AND (Intervertebral Disc Degeneration) showed 2 results. All these results together totaled 26, with 9 articles being discarded as out of topic, 9 articles as reviews and 1 article was duplicated.

**Figure 3:**
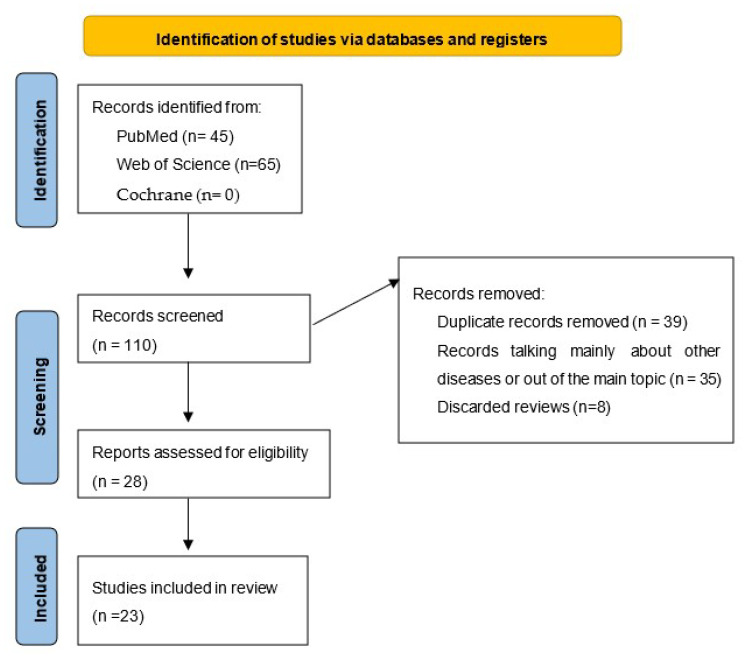
PRISMA 2020 Flow chart. This figure synthetizes the several phases of this systematic review. The data were collected from August to September 2022 and reviewed in September and October.

In Web of Science (WOS), we found the following results searching only as document type: article and discarding reviews and other type of texts: Progranulin in Rheumatoid Arthritis 33 results: 10 of them were out of our main topic, 17 of them were duplicated. Progranulin in Lupus Erythematosus: 16 results, 7 of them out of the main topic and 8 from them were duplicated. Progranulin in Intervertebral Disc Degeneration: 7 results, 3 were out of topic and 4 duplicated. Atsttrin in Rheumatoid Arthritis: 3 results. Atsttrin in Lupus Erythematosus: 0 results. Atsttrin in Intervertebral Disc Degeneration: 2 results (see Figure 3). In this last two searches, all of them were discarded because were duplicated and included in the PubMed search.

The results taken in this review are mainly descriptive with clear focus on the state of art of the adipokine itself, and are not clinical trial or meta-analysis studies, for that we did not consider determining the risk of bias.

### 2.1. Progranulin in Rheumatoid Arthritis

Rheumatoid Arthritis is a common disorder that affects a lot of population all around the world. In 2019, 14 million cases of RA were reported [28]. It is a systemic autoimmune disorder in which joints get swollen, warm and painful. The complete mechanism of action of this disease is still undescribed, but inflammation towards TNF-α is supposed to be involved, in fact, inhibitors of this pathway are among the most effective treatments. As previously described, PGRN is an antagonist of endogenous TNF-α [29]; therefore, this adipokine could be a therapeutic target in RA.

**Figure 4:**
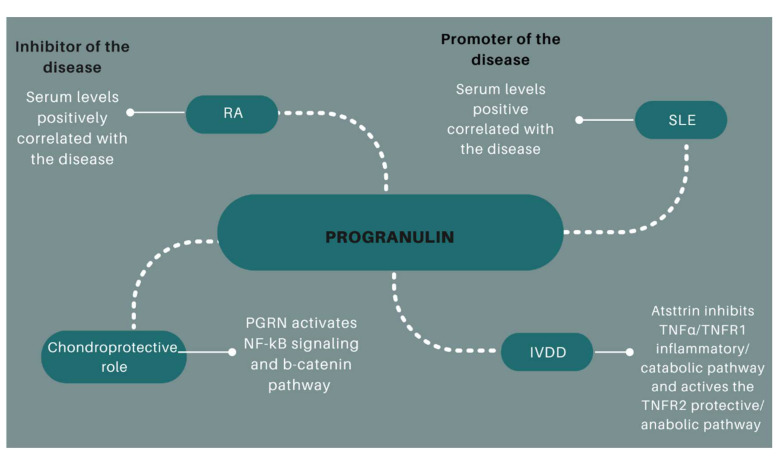
Schematic representation of PGRN relevance in RA, SLE and IVDD.

PGRN’s function in RA inflammation is not yet entirely understood. TNF-mediated inflammation can be avoided by PGRN’s ability to block intracellular signaling that is induced by TNF-α. TNF-α levels in serum, which is connected to the severity of the disease in RA patients, were found to be strongly correlated with the serum PGRN level (Figure 4) (Table 1) [30]. The group of Tang et al. reported that PGRN binds to TNFR and interrupts TNF-α/TNFR interaction [19]. PGRN characteristics have been studied in PGRN knock out mice model with collagen-induced arthritis (CIA), determining that these mice developed higher risk of RA and joint destruction compared to controls [31,32]. The administration of PGRN in these mice reverted inflammatory arthritis [19]. PGRN also takes part in the conversion of Tregs, which are immune-suppressive in autoimmune inflammatory diseases [30,33].

The presence of anti-PGRN antibodies was described to be correlated to autoimmune diseases [34]. The determination of serum PGRN-abs for use as a novel diagnostic marker in the standard serological diagnostic for RA, alongside rheumatoid factor (RF) and anticitrullinated protein antibody (ACPA), should be explored in light of this specificity of serum PGRN-abs for autoimmune disorders [35].

Higher levels of PGRN were detected in serum and synovial fluids of RA patients compared to OA patients [23,29], being correlated with disease activity and with ultrasound evaluation [36,37]. Other studies suggest that PGRN can play a role in miR-138 and histone deacetylase 4, affecting NF-kB pathway in RA [38].

A prospective and observational study investigated PGRN as a possible predictor to the response to TNF-antagonist therapy, finding that pretreatment levels of this adipokine were not correlated to the response to the treatment [39]. However, after starting the treatment, serum PGRN levels were associated with erythrocyte sedimentation rate (ESR), C-reactive protein (CRP), Disease Activity Score 28-joint count C reactive protein (DAS-28-CRP), Disease Activity Score 28-joint count with erythrocyte sedimentation rate (DAS-28-ESR) and Clinical Disease Activity Index (CDAI). In this context, patients with the highest PGRN levels had a better response. Johnson et al. determined that PGRN is not a good predictor for the TNF-antagonist therapy, but the administration of PGRN might represent a valid therapeutic option due to its activity as TNF-antagonist [39].

Wang et al., have described that in a mesenchymal stem cell line (C2C12), PGRN can decrease the TNF-α in an osteoblast differentiation-induced model, playing a protective role [40]. PGRN also demonstrated rescue role in the TNF-α-induced cartilage oligomeric matrix protein (COMP) degradation [40,41]

As a proteolytic degradation disease, arthritis can affect the cartilage producing loss of extracellular matrix components. Recently, it was described that PGRN binds to ADAM Metallopeptidase with Thrombospondin Type 1 Motifs 7 and 12 (ADAMTS-7 and ADAMTS-12) and metalloproteinases that contribute to cartilage extracellular matrix degradation. Through this mechanism, PGRN can make a positive effect in terms of prevention and treatment of cartilage degeneration [42].

### 2.2. Progranulin in Systemic Lupus Erythematosus

Systemic lupus erythematosus (SLE) is a multisystem autoimmune disease that affects mainly women between puberty and menopause. The etiology of the disease is still unclear. Nevertheless, genetic, environmental and hormonal factors are thought to be involved in the pathogenesis and the progression of the disease [43]. SLE is a vastly heterogenous disease where patients present diverse autoantibodies and different organ involvement which hamper understanding the disease [44]. The disease is characterized by the breakdown of tolerance to self-antigens and following autoantibody production that causes chronic inflammation. The immune system can further attack and destroy different organs including the joints, the central nervous system, and the kidneys.

In this context, increased plasma PGRN levels have been reported in SLE patients in comparison to controls [45,46] (Table 2) (Figure 4). Furthermore, its levels were decreased after the treatment and the improvement of the disease. Accordingly, Tanaka et al. reported that serum levels were directly correlated with SLE Disease Activity index and anti-doublestranded DNA antibody (anti-dsDNA) and inversely correlated with CH50, C3, C4 levels [45]. Taken together, these data suggest the PGRN as a biomarker reflecting the SLE disease activity [45].

Jing et al. used a SLE pristane-induced mice model to determine the PGRN role in pathogenesis of SLE. In this model, PGRN knock out mice seemed to be less damaged by the SLE-induced model than those wildtypes. Furthermore, anti-ribosomal protein P0 and anti-dsDNA levels in serum were lower in PGRN-/- SLE than healthy controls [46]. All these data support the idea that PGRN levels were highly correlated with the severity of the disease [46]. However, some discrepancies have been postulated. Chougule et al. determined that elevated PGRN levels were not correlated with the severity of the disease, but they were with the antiribosomal P0 antibodies [47].

**Table 2:** Main findings related to PGRN in SLE.

Subjects	Study	Ref.
SLE patients	Levels of PGRN are higher in SLE	[45,46]
SLE patients	Levels of PGRN in SLE patients are higher and correlated to anti-dsDNA antibodies. Also correlated with proinflammatory cytokinesPGRN downregulated with glucocorticoids	[48]
PGRN -/- mice model	Significant decrease in anti-dsDNA serum levels in PGRN-/- SLE mice compared with controls	[46]
SLE patients	PGRN levels correlated with antiribosomal P0 antibodies	[47]

PGRN deficient mice showed lower levels of Th1 and Th17 compared to controls, but in contrast, Th2 and Treg were higher in the spleen, which contributes to a downregulation of inflammatory cytokines such as Interferon gamma (IFNγ) or Interleukin-17A and an upregulation of anti-inflammatory cytokines (IL-4 and IL-10) [46].

In clinical terms, PGRN was reported to be downregulated after the administration of high doses of glucocorticoids, in particular prednisone [48]. Based on the literature, PGRN’s role in this disease has not been deeply studied and the mechanism is not clearly described; therefore, further studies are needed to determine its functions.

### 2.3. Progranulin in Intervertebral Disc Degeneration

Intervertebral disc degeneration (IVDD) is a chronic musculoskeletal disease characterized by a degenerative cascade causing progressive inflammatory, metabolic, and structural changes in the IVD. This degeneration process is accompanied by a persistent low-grade inflammation sustained by the production of higher levels of proinflammatory adipokines and cytokines [49]. The persistent low-grade inflammation causes extracellular matrix degradation, fibrosis, loss of mechanical stability and shock absorbent function of the IVD, and further damage of the intervertebral tissues [50]. TNFα is widely accepted to be involved in the pathogenesis of IVDD [51]. Being an antagonist of TNFα/TNFR signaling, PGRN could be a potential candidate for modulating IVDD [31].

Wang et al. reported elevated serum PGRN levels and higher expression of PGRN in the disc tissues of IVDD patients compared to controls [51]. The same study also suggested a protective role of PGRN in IVDD by inducing the production of the anti-inflammatory cytokine IL-10 and inhibiting the proinflammatory TNFα-induced IL-17. Zhao et al. have described that PGRN-/- mice increased IVDD progression, accompanied with abnormal bone formation and high resorption. The mechanism of these processes could be the activation of NF-kB signaling and b-catenin pathway (Figure 4) [52]. With these preliminary results we highlight the importance of increasing the knowledge of PGRN as a potential therapeutic target for IVDD.

### 2.4. Atsttrin

Atsttrin is an engineered molecule derivate from PGRN. It is formed of the granulin units F, A and C, and the linkers, P3, P4 and P5. With this changes, this molecule loses the activity relative to oncogenesis, as PGRN has [19], while retaining the affinity to the TNF receptors [22]. Another significant difference between Atsttrin and PGRN is the half-life time being 120 and 40 h, respectively. This makes Atsttrin more interesting as a potential treatment molecule [22].

Multiple animal models of arthritis have demonstrated that Atsttrin has a protective role in chondrocytes and stimulates chondrogenesis [19,53,54]. Cartilage recovery with Atsttrin treatment was described to be dependent on the presence of TNFR2, because the capacity of repair in the TNFR2-/- model mice was significantly reduced compared with TNFR1-/- [55]. Atsttrin was described to bind with TNFR2 and activate Akt signaling and JunB, promoting cartilage recovery [55]. It has also been used as an improving factor in 3D printed scaffolds, contributing to bone repair thought TNF pathway [56].

In addition, Atsttrin was described as a more protecting molecule in inflammatory arthritis than PGRN [19,57]. However, regarding OA development, there was no significant difference between these two molecules [54].

TNF-α has been reported to be upregulated in IVDD. In this context, Atsttrin can play an important role in the treatment of this disease inhibiting the TNF-α-induced nucleus catabolism and reducing inflammatory cytokines (Figure 4) [58,59].

All these findings suggest that Atsttrin has an important role in the recovery and improvement of degenerative joint diseases and conforming a potential therapeutic treatment.

## 3. Discussion

This review shows evidence supporting the implication of PGRN in musculoskeletal inflammatory and degenerative disorders, although many mechanisms are still elusive. Nevertheless, the antagonism of PGRN to TNFα seems a key mechanism to understand this implication [19]. In RA and IVDD, the results presented in this review indicate an anti-inflammatory and chondroprotective roles of PGRN. In addition, PGRN was shown to be a biomarker indicating disease progression. This is in line with the previously described anti-inflammatory activity of PGRN in several conditions. PGRN was reported to ameliorate brain inflammation and reduce neutrophil infiltration, leading to crucial brain protective role in patients suffered from cerebral ischemia-reperfusion (I/R) injury [60,61]. In addition, PGRN showed an anti-inflammatory and protective role in renal and lung injury mouse models [62,63,64].

On the other hand, available studies on the role of PGRN in SLE point out a proinflammatory implication in this disease. Taking into account that SLE is a multisystem disease, it is worth mentioning that PGRN was recently associated with worsening systemic inflammation, acting at the same time locally as renal anti-inflammatory [65]. Nevertheless, the mechanism of the involvement of PGRN in SLE is not well defined yet, and the published studies are very limited. An interesting perspective would be to explore whether any further cleavage of PGRN is causing this proinflammatory activity mediated by granulins in SLE [66].

As reported in this review, describing the binding domains in PGRN was a milestone for identifying new therapeutic targets in diseases such as RA. The identification of the PGRN/TNFR binding domains was also crucial for designing Atsttrin, the new engineered molecule with higher stability than PGRN. This substance, as described in this review, has more specific anti-inflammatory and regenerative properties in chondrocytes than PGRN.

In 2013, PGRN was discovered to also be an autoantigen, which can normally be a target of autoantibodies in the serum in patients with vasculitis [34]. These antibodies are not specific for a particular autoimmune disease, but they are for autoimmune processes. It is particularly intriguing that PGRN antibodies levels are strongly correlated with reduced plasma levels of PGRN, indicating that PGRN could be neutralized by these antibodies. The characterization of the autoantibodies profile in different patients with autoimmune diseases can have an important impact in the search of new treatments. Human recombinant PGRN and Atsttrin are considered a new generation of TNF-blockers [67], which could be a new therapeutic strategy if we assumed that patients with anti-PGRN antibodies produce an overexpression of TNFR1 and 2.

This review shed light on the limited available publications regarding the role of PGRN in musculoskeletal inflammatory disorders. Being an antagonist to such a master inflammatory cytokine as TNF-α, PGRN seems to be a promising therapeutic target for modulating several auto-immune diseases. More research is needed to understand the role of PGRN in musculoskeletal inflammatory disorders and to develop novel pharmacological approaches for the treatment of these conditions.

## 4. Materials and Methods

This systematic review follows the procedure of the PRISMA statement [27]. We conducted a search on PubMed (MEDLINE) based on the terms and the data. We made 3 searches with the terms combinations (Progranulin) AND (Lupus Erythematosus, Systemic), (Progranulin) AND (Arthritis, Rheumatoid), and (Progranulin) AND (Intervertebral Disc Degeneration). The Medical Subject Headings were not included because they did not include progranulin. On the other hand, we examined with the terms combinations (Atsttrin) AND (Lupus Erythematosus, Systemic), (Atsttrin) AND (Arthritis, Rheumatoid), and (Atsttrin) AND (Intervertebral Disc Degeneration).

In addition, we performed 6 searches in Web of Science (WOS), Progranulin in Rheumatoid Arthritis, Progranulin in Lupus Erythematosus, Progranulin in Intervertebral Disc Degeneration, Atsttrin in Rheumatoid Arthritis, Atsttrin in Lupus Erythematosus and Atsttrin in Intervertebral Disc Degeneration, filtering with: Refined By: Document Types: Article; NOT Document Types: Review Article.

The same searches were performed in Cochrane Library, finding no results; therefore, the studies included in this review were limited to the results obtained from two databases: PubMed and WOS.

Due to the limited number of studies found, we did not filter the results based on the year of publication. All these sources were searched in September 2022.

The inclusion criteria were the studies whose main topic were Progranulin, or Atsttrin, with emphasis in the three selected diseases. All the studies we included were written in English. Second, the exclusion criteria were studies which were not written in English and those only focused on diseases not related to RA, lupus or IVDD, and the literature reviews.

## Figures and Tables

**Table 1:** Main findings in studies of PGRN in RA.

Subjects	Study	Ref.
PGRN -/- mice	The administration of PGRN in these mice reverts inflammatory arthritis	[19]
RA patients	PGRN serum and synovial fluids levels in RA patients are higher compared to controls	[23,29]
RA patients	PGRN serum levels higher in patients with high disease activity compared with low to moderate activity.	[30]
RA patients	PGRN levels associated with RA activity	[36]
RA patients	PGRN importance as a biomarker for RA	[37]
RA patients	Time-dependent changes in RA parameters are correlated with blood PGRN levels	[39]
Mesenchymal stem cell line (C2C12)	PGRN decreased TNF-α in an osteoblast differentiation-induced model	[40]
Inflammatory arthritis mice model	PGRN decreases COMP fragments	[41]
Human cells	PGRN binds to ADAMTS-7 and -12 preventing cartilage degeneration	[42]

## Data Availability

Data sharing not applicable.
